# Increasing incidence of syphilis among patients engaged in HIV care in Alberta, Canada: a retrospective clinic-based cohort study

**DOI:** 10.1186/s12879-018-3038-4

**Published:** 2018-03-13

**Authors:** Raynell Lang, Ron Read, Hartmut B. Krentz, Soheil Ramazani, Mingkai Peng, Jennifer Gratrix, M. John Gill

**Affiliations:** 1 Department of Medicine, University of Calgary, S Alberta HIV Clinic, 3330 Hospital Drive NW, Calgary, AB T2N4N1 Canada; 2 S Alberta HIV Clinic, Calgary, Canada; 3 Department of Community Health Sciences, University of Calgary, Calgary, Canada; 4 Alberta Health Services STI Centralized Services, Edmonton, Canada

**Keywords:** Syphilis, HIV/AIDS, Incidence, Canada

## Abstract

**Background:**

Syphilis is a global health concern disproportionately affecting HIV-infected populations. In Alberta, Canada, the incidence of syphilis in the general population has recently doubled with 25% of these infections occurring in HIV-infected patients. The Southern Alberta HIV Clinic (SAC) and Calgary STI Program (CSTI) analyzed the epidemiologic characteristics of incident syphilis infections in our well-defined, HIV-infected population over 11 years.

**Methods:**

Since 2006, as routine practice of both the Southern Alberta Clinic (SAC) and Calgary STI Programs (CSTI), syphilis screening has accompanied HIV viral load measures every four months. All records of patients who, while in HIV care, either converted from being syphilis seronegative to a confirmed seropositive or were re-infected as evidenced by a four-fold increase in rapid plasma reagin (RPR) after past successful treatment, were reviewed.

**Results:**

Incident syphilis was identified 249 times in 194 HIV-infected individuals. There were 36 individuals with repeated infections (28.5% of episodes). Following a prior decline in annual incident syphilis rates, the rates have tripled from 8.08/1000 patient-years (95% confidence interval (CI): 4.14–14.75) in 2011, to 27.04 per 1000 person-years (95% CI: 19.45–36.76) in 2016. Half of the syphilis episodes were asymptomatic. Patients diagnosed with syphilis were twice as likely not to be taking ART and had a higher likelihood of having plasma HIV RNA viral loads > 1000 copies/mL (19%). Incident syphilis was seen predominantly in Caucasians (72%, *P* < 0.001), males (94%, *P* < 0.001) and men who have sex with men (MSM) as their HIV risk activity (75%, *P* < 0.001).

**Conclusions:**

We have highlighted the importance of a regular syphilis screening program in HIV-infected individuals demonstrated by increasing rates of incident syphilis in our region. Targeted preventative strategies should be directed towards HIV-infected populations identified at highest risk, including; MSM, prior alcohol abuse, prior recreational drug use and those with prior syphilis diagnoses.

**Electronic supplementary material:**

The online version of this article (10.1186/s12879-018-3038-4) contains supplementary material, which is available to authorized users.

## Background

The global reemergence of syphilis as a common and clinically significant sexually transmitted infection (STI) is forcing healthcare providers to search for the new elements driving this epidemic. It is estimated that worldwide, there are nearly twelve million new infections with syphilis annually [1]. Syphilis rates in Canada more than doubled between 2003 (2.9/100,000) and 2012 (5.8/100,000), with the rates increasing among females by 40.9% and in males by 128.3% [2]. In the Canadian province of Alberta, the rates of new cases of syphilis increased dramatically between 2014 and 2015 nearly doubling in number, with over 25% of cases occurring in patients co-infected with HIV [3].

Syphilis, similar to other STI’s, acts synergistically with HIV, both increasing susceptibility to infection and leading to worse health outcomes [4–6]. Asymptomatic or undiagnosed syphilis negatively impacts HIV prognoses by reducing CD4 counts and increasing viral loads making early syphilis detection important when delivering HIV care [5–8]. Recent studies have suggested that HIV-infected patients, while receiving consistent HIV care, may continue to engage in sexual activities placing themselves at high risk for acquiring other STI’s [9, 10]. The suppression of HIV viral replication (HIV viral load < 1000 copies/mL) using antiretroviral therapy (ART) resulting in minimal risk for sexual transmission of HIV has received legal recognition in Canada [11–15].

This increase in episodes of syphilis being seen in southern Alberta prompted the Calgary STI Program (CSTI) and the Southern Alberta Clinic (SAC), to coordinate their initiatives to improve syphilis screening in those engaged in HIV care. In this retrospective cohort study, we aimed to characterize incident syphilis episodes in HIV-infected individuals, assessing patient demographics and markers of HIV control. Such knowledge may be essential for developing improved and targeted preventative strategies especially for patients engaged in ongoing HIV care.

## Methods

### Study population

SAC and CSTI are the sole referral agencies for both syphilis and HIV within the region, facilitating inclusivity of this retrospective cohort study. SAC provides HIV care to all HIV-infected individuals living within southern Alberta, Canada and CSTI provides all health care services for STI’s from testing to treatment.

In a quality assurance project (approved by University of Calgary Bioethics committee) at both SAC and CSTI programs, routine serologic screening for syphilis regardless of risk accompanied regular four-month HIV viral load measurements between January 1, 2006 and December 31, 2016. The records of all episodes of incident syphilis infection occurring in HIV-infected patients were reviewed. Every indeterminate or positive syphilis serology for a SAC patient was discussed with or referred to CSTI at the time of testing.

All individuals followed at SAC with at least one regular visit between January 1, 2006 and December 31, 2016 were included in this study (*N* = 2448). Patients were followed until December 31, 2016 or until they either moved, died or were lost-to-follow-up. All patients, who while in HIV care, converted from being seronegative for syphilis to a confirmed positive status or were re-infected with syphilis were reviewed through the SAC database and a CSTI chart review. A control population was identified through the SAC database to be used for comparison of demographic data and included all HIV-infected patients followed at SAC with at least one regular visit between January 1, 2006 and December 31, 2016 whose treponemal or non treponemal syphilis test remained negative.

### Syphilis diagnosis

The syphilis screening algorithm and confirmatory testing was achieved using indirect serologic methods. Initially screening for syphilis was done with the non-treponemal rapid plasma reagin (RPR), however in 2008 the screening test was changed to an enzyme immunoassay (EIA), a treponemal test. The RPR continued to be used as a confirmatory test as well as for monitoring response to therapy. In Calgary, the secondary confirmatory test was either the fluorescent treponemal antibody absorption test (FTA-ABS) or the line immunoassay (INNO-LIA). Repeat syphilis episodes were identified by a four-fold increase in RPR after a prior documented successful treatment course for syphilis and were evaluated and staged by an STI specialist (RR).

### Data collection

Clinical and sociodemographic data was obtained from a review of both SAC and CSTI databases and charts for all patients included in the study. These data included: age at HIV diagnosis; gender (i.e. male, female, transgendered), self-reported ethnicity (i.e. Caucasian, Indigenous, African/Caribbean/Black (ACB), Other); most likely HIV exposure risk (i.e. MSM-self-reported men who have sex with men identification, HET- self-reported heterosexual identification, PWID- self-reported persons who inject drugs, Other), and year of HIV diagnosis. Other self-reported data fields included cigarette smoking (current or past), alcohol abuse (> 14 drinks/week or self-described binge drinking), recreational drug use (i.e. intravenous drug use), intimate partner violence exposure (current, past and/or childhood abuse), and ever unstable housing (i.e. homelessness, transitional housing, shelters, supported housing).

For patients with an incident syphilis infection, detailed standardized information was collected by one physician (RL), through a comprehensive review of both SAC and CSTI charts and databases. Multiple sources including nursing interviews, social work reports, self-administered questionnaires, laboratory reports and routine STI and HIV care notes from charts and databases were used. These data included self-described sexual practices, ART use and HIV viral loads collected both prior to and after each positive syphilis test. Prior history of comorbid infections including *Neisseria gonorrhoeae*, *Chlamydia trachomatis* and Hepatitis C virus (HCV*)* were self-reported and documented in CSTI charts at the time of syphilis diagnosis.

During routine HIV appointments, all patients were asked if they were sexually active and if the activities would be considered “unsafe” (condomless sex). For those engaging in unsafe sexual practices, counselling on both HIV and STI transmission risks was provided. The number of self-reported sexual partners in the period six months prior to testing positive for syphilis was documented. All data was anonymized prior to analysis.

### Statistical analysis

Demographic and risk factors of patients in control group were compared with those with syphilis infection, those with repeat episodes of syphilis, those not on ART, and those not virally suppressed (viral load > 40 copies/ml) using chi-squared testing. Age and sex adjusted incident rate (IR) of syphilis infection were calculated from 2006 to 2016 using the 2006 demographic as reference. Incidence rates adjusted for HIV risk exposure in addition to age and sex were also analyzed. Incidence rates were calculated using patients “active in care,” meaning they had attended at least one SAC visit each year to contribute to a patient-year of follow up. All statistical analysis was performed using Stata, (version 15.0 College Station, TX). All charts were created with Microsoft Excel.

## Results

Between 1/1/2006 and 12/31/2016, the mean number of patients “active in care” at SAC was 1517 per year (range: 1086 in 2006 to 1889 in 2016). During this time, there were 20,203 syphilis tests done on a total of 2448 patients who attended at least one regular SAC visit. On average, there were 180 days between each syphilis test per patient. The average number of syphilis screening tests that were done per patient each year over the 11-year period was 2.1. In 2006 the average number of tests per year was 1.3, whereas in 2016 this was 2.8. For high risk patients (MSM) screening rates were more frequent with the average testing over 11 years being 2.4 tests per year. The average number of viral load measurements per patient per year was 3.03 (range 2.58–3.37).

Of the 2448 HIV-infected patients followed at SAC and CSTI during the study period, encompassing 15,175 person-years, we identified 360 potential episodes of syphilis in 305 different patients that met the broad study criteria for incident syphilis. Of the 360 potential episodes, 38 were excluded after being classified as false positive syphilis tests. Inadequate availability of comprehensive data led to exclusion of 32 episodes along with 41 additional episodes, who although tested in Alberta, had since moved out of province. These episodes were not included in incident rate calculations as validity of the tests ensuring incident infection as well as characteristics of the diagnosis were not available. We therefore analyzed 249 episodes in 194 individuals. Over half (50.8%) of incident syphilis cases were asymptomatic and were only identified by routine screening. In 71/249 (28.5%) of the infections, syphilis occurred in individuals who had previously been successfully treated for syphilis on one or more past occasions and were identified by a four-fold increase in RPR titer.

**Fig. 1:**
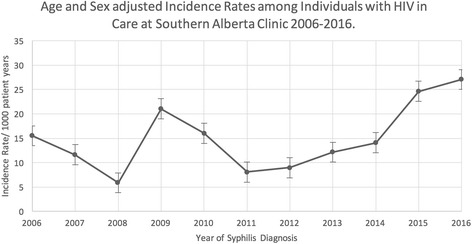
Age and Sex adjusted Incidence Rates among Individuals with HIV in Care at Southern Alberta Clinic 2006–2016. This figure demonstrates the rise in incident rate per 1000 patient years of syphilis infection in the HIV-infected individuals at SAC between 2006 and 2016

**Fig. 2:**
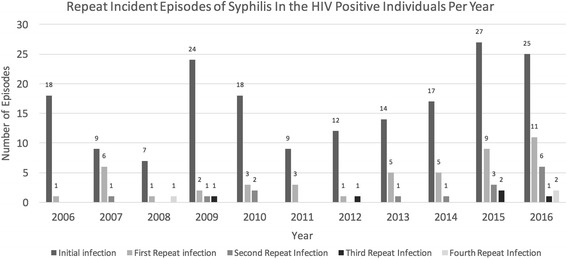
Repeat Incident Episodes of Syphilis in the HIV Positive Individuals Per Year. This figure is demonstrating the number of syphilis infection each year divided by number of repeat infection. As seen there has been an increase in number of syphilis episodes from 2006 to 2016, however the number of repeat cases has also increased

The average IR between 2006 and 2016 was 12.88 per 1000 person-years of follow up (95% confidence interval (CI): 9.64–16.11). When adjusted for HIV exposure category in addition to age and sex, the average IR between 2006 and 2016 was 13.22 per 1000 person-years of follow up (95% CI: 8.20–21.34). Incident rates decreased from 15.50 in 2006 (95% CI: 9.04–26.35) to 5.87 in 2008 (95% CI: 2.68–12.77). The rise in syphilis rates in 2009 (Fig. 1) are likely reflective in part to a change in the testing algorithm for syphilis in Calgary from an initial RPR to enzyme immunoassay (EIA). In 2008, the screening test was changed to an EIA resulting in increased identification of latent syphilis due to improved sensitivity of the test and likely resulting in the increasing rate of syphilis demonstrated in 2009 and 2010. Annual incidence rates tripled from 2011 8.08/1000 patient-years (95% CI: 4.14–14.75), to 27.04 per 1000 person-years (95% CI: 19.45–36.76) in 2016 (Fig. 1). The number of repeat syphilis infections climbed from three repeat episodes in 2011 (25% of all syphilis infections during that year), up to 20 in 2016 (44%) (Fig. 2).

### Demographics

The incident syphilis infections were seen mainly in males (94%) compared to the control group (74%, *P* = < 0.001), with MSM being the leading HIV exposure risk group (75% vs 40.6%, *P* = < 0.001) and in Caucasians (72% vs. 56%), *P* = < 0.001). Compared to HIV-infected but with no evidence of past or current syphilis infection, those with syphilis were more likely to have a history of alcohol abuse (28% vs. 18%, *P* = < 0.001) and a history of recreational drug use (44% vs. 31%, *P* = < 0.001) (Table 1). Co-infections with other STI’s (*C. trachomatis, N. gonorrhoeae,* HCV*)* at time of syphilis infection were very common with 42% of the population studied having had at least one other STI prior to incident syphilis infection other than HIV. STI co-infections included; 32% of cases having a prior history of *N. gonorrhoeae*, 24% having had documented *C. trachomatis* and 1.5% were also co-infected with HCV.

**Table 1:** Characteristics of HIV+ patients regularly followed at the Southern Alberta Clinic between 1/1/2006 and 12/31/2016 comparing patients who were negative for syphilis (Syphilis Neg) to patients who ever tested positive for syphilis (Syphilis Pos)

	Total	Syphilis Neg	Syphilis Pos	*P*-value
N (%)	2448	2254 (92.1)	194 (7.9)	
Age at HIV Diagnosis (years)				
Mean (range)	35 (1–79)	35 (1–79)	35 (16–69)	0.893
< 30	888 (36.2)	813 (36.1)	75 (38.7)	0.801
30–39	868 (35.5)	802 (35.6)	66 (34.0)	
40–49	475 (19.4)	438 (19.4)	37 (19.1)	
≥ 50	217 (8.9)	201 (8.9)	16 (8.3)	
Gender				
Male	1858 (75.9)	1675 (74.3)	183 (94.3)	<.001
Female	583 (23.8)	572 (25.4)	11 (5.6)	
Transgendered	7 (0.3)	7 (0.3)	0 (0.0)	
Self-reported Ethnicity^a^				
Caucasian	1399 (57.1)	1259 (56.0)	140 (72.2)	<.001
Indigenous	222 (9.1)	216 (9.6)	6 (3.1)	
ACB	560 (22.9)	536 (23.8)	24 (12.4)	
Other	267 (10.9)	243 (10.8)	24 (12.4)	
Most Likely HIV Exposure Category^b^				
MSM	1060 (43.3)	915 (40.6)	145 (74.4)	<.001
HET	526 (21.5)	512 (22.7)	14 (7.2)	
PWID	761 (31.1)	731 (32.4)	30 (15.6)	
Other	101 (4.1)	96 (4.3)	5 (2.6)	
Co-factors^c^				
History of Smoking	1252 (51.1)	1142 (50.7)	110 (56.7)	0.124
History of Alcohol Abuse	460 (18.8)	404 (17.9)	56 (28.9)	<.001
History of Recreational				
Drug Use	794 (32.4)	707 (31.4)	87 (44.8)	<.001
History of Unstable				
Housing	516/2109	483/1932	33/177	0.073
	(24.5)	(25.0)	(18.6)	
History of Intimate				
Partner Violence	655 (26.7)	614 (27.2)	41 (21.1)	0.079

^a^Native Canadian includes Aboriginal, Metis and Inuit; ACB includes African, Caribbean, Black; Other includes IndoAsian, Hispanic, East Asian, and other

^b^MSM = self-reported men who have sex with men identification; HET = self-reported heterosexual identification; PWID = self-reported intravenous drug use identification; Other HIV Risk factor behavior includes: blood transfusions, hemophiliac, neonatal, postnatal infection, unknown or not reported

^c^History of Cigarette Smoking-Current or Past; History of Alcohol Abuse- > 14 drinks/week or binge drinking; History of Recreational Drug Use –Current or Past; History of Unstable Housing-homelessness, shelters, supporatative housing; History of Intimate Partner Violence-Current or Past

### Engagement with HIV care and disclosure of sexual activity

The mean age at syphilis diagnosis was 47 years (range of 21–72); the mean length of time between the HIV positive test and a positive syphilis test was 7.7 years (range 0.1–25.6) with 31% having been HIV positive for greater than 10 years (Table 2). The mean duration of HIV care at SAC was 4.9 years (0.1–23.3) with 16% having been followed for greater than 10 years. At the visit prior to acquiring syphilis, only 59% stated that they were currently sexually active; however, following diagnosis with syphilis, 71% reported sexual activity. There were 144/193 (75%) patients diagnosed with syphilis that reported having 1 to 5 sexual partners in the preceding 6 months of their syphilis diagnosis; mean number of partners was 4.7 and median being 2. Counselling on safer sexual practices was provided for 44% of the individuals at the clinic visit immediately prior to testing positive for syphilis and 50% received safer sex counselling at the appointment immediately following syphilis infection.

**Table 2:** Characteristics of HIV+ patients active in care at SAC or CSTI at time of their syphilis diagnosis between 1/1/2006 and 12/31/2016. (Note − 158 patients had a single syphilis episode; 36 patients had more than 1 episode. *N* = 249 syphilis episodes total)

Length of time in years between HIV diagnosis and Syphilis diagnosis (*n* = 249)	
Mean (range)	7.7 (0.1–25.6)
< 1 year (N/%)	26 (10)
1–5 years	82 (33)
6–10	64 (26)
> 10 years	77 (31)
Age in years at time of each Syphilis diagnosis (*n* = 249)	
Mean (range)	47 (21–72)
< 30 (N/%)	43 (17)
30–39	74 (30)
40–49	81 (33)
50–59	37 (15)
> 59	14 (6)
Length of time in years followed in care at SAC (*n* = 249)	
Mean (range)	4.9 (0.1–23.3)
< 1 (N/%)	77 (31)
1–5	101 (41)
6–10	31 (12)
11–15	24 (10)
> 15	16 (6)
Number of self-reported sexual partners within the preceding 6 months of a syphilis diagnosis (*n* = 193)	
Mean (range)	4.7 (0–50)
0 (N/%)	14 (7)
1–5	144 (75)
6–10	15 (8)
11–15	6 (3)
16–20	8 (4)
> 20	6 (3)

### HIV control at syphilis diagnosis

At the time of their incident syphilis infection, nearly 1 in 5 (19%) of patients were not on ART in contrast to 11% of other HIV-infected patients in care in the same time period. Those not on ART with incident syphilis were more likely to be PWID (23%), compared to those on ART (16%, *P* = < 0.004). Self-reported alcohol abuse was also more common in those not on ART (33% vs. 29%, *P* = 0.024) (Additional file 1: Table S1).

At the time of syphilis infection, nearly one-third (30.5%; 75/249) of patients had detectable HIV viremia (ranging from 49 to 2,309,021 copies/mL) (Table 3). This included 19% of patients that had viral loads greater than 1000 copies/mL and 4% had viral loads of greater than 100,000 copies/mL. In patients with viral loads > 40 copies/mL at syphilis diagnosis, 52% were not on ART. These patients with detectable viremia were predominately male (96%, *P* = 0.002), Caucasian (77%, *P* = 0.04) and MSM (73%, *P* = < 0.001) and a greater proportion were PWID (20%, *P* = < 0.001) (Additional file 1: Table S1).

**Table 3:** Subgroup analysis of individuals with HIV and incident syphilis infection based on virologic supression^a^, patients not on antiretroviral therapy (ART) and those with repeat syphilis episodes

Episodes of syphilis in individuals with HIV that were not virologically suppressed, VL ≤ 40 copies/mL (*n* = 75/249)	Number of Episodes	Percentage
On ART	36	48
Not on ART	39	52
Episodes of syphilis in individuals with HIV that are not on ART (*n* = 48/249)	Number of Episodes	Percentage
Viral Load (range) copies/mL	< 40–2,309,021	
≤ 40	2	4
41–399	4	8
400–10,000	18	38
≥ 10,000	24	50
Episodes of syphilis in individuals with HIV and repeat syphilis infections (*n* = 71/249)	Number of Episodes	Percentage
Virologic suppression (range)	< 40–330,000	
Virologically suppressed	45	63
Not Virologically suppressed	26	37
On ART	58	82
Not on ART	13	18

^a^HIV Virologic suppression defined as < 40 copies/mL

### Subgroup analysis: Repeat episodes of syphilis

Over one-quarter (28%) of syphilis infections occurred in individuals previously successfully treated for a past episode and were identified by a four-fold increase in RPR. Over two-thirds (47/71) were second episodes of syphilis, with 21% (15/71) experiencing syphilis for the third time, 9% (6/71) for the fourth time and 4% (3 individuals) for the fifth time. Those experiencing repeat episodes of syphilis were more likely male (97%, *P* = 0.007), Caucasian (75%, *P* = 0.001) and MSM (92%, *P* = < 0.001) compared to those in the control population of HIV-infected, syphilis negative individuals. A trend towards current or past recreational drug use (47%, *P* = 0.064) was noted but not reaching significance in those with repeat infections (Additional file 1: Table S1). A history of other STI’s were very common in this subgroup with a prior history of *N. gonorrhoeae* (47%) and *C. trachomatis* (39%).

## Discussion

In many countries, the rate of new syphilis infections in HIV-infected patients is increasing [16, 17]. We found that the annual incidence rate of new syphilis infections in patients regularly engaged in HIV care at SAC has nearly tripled since 2011. Overall, 8% of all SAC patients have experienced one or more syphilis episodes during the past 10 years; 1 in 7 (13.5%) MSM individuals at SAC have been diagnosed with syphilis. Over half (50.8%) of the new infections were asymptomatic and only identified by routine screening during HIV care, underscoring the importance and value in routine screening in this risk population. Over 1 in 4 (28%) of previously diagnosed patients with syphilis had one or more subsequent syphilis episodes.

These results are disconcerting as patients in this study had been successfully engaged in care for an average of 5 years with over 15% followed for 10 years or more. Patients routinely are screened for syphilis approximately every 4 months, and are counselled on safe sex practices on an ongoing basis. By routinely screening patients we have likely identified increased numbers of asymptomatic syphilis episodes that were diagnosed, however this does not explain the increasing number of incident infections and our failure in curtailing this epidemic.

STI prevention may be achieved through counselling and education [18–20]. Flemming et al. stated that three key areas need to be addressed in programmatic developments addressing STI infections in HIV-infected populations including improving access to clinical services, promoting early and effective STI related healthcare behaviors and lastly, establishing successful screening systems [21]. To be cost effective, novel programing must be focused towards those at greatest risk of incident syphilis infection. High risk groups identified for acquiring syphilis among HIV-infected populations include MSM, current or prior alcohol abuse disorder, history of recreational drug use and prior syphilis diagnosis.

As in other reports, men accounted for most of our new syphilis infections with the majority having MSM as their risk activity for their HIV [3, 17]. The successful use of ART in almost eliminating HIV transmission risk in adherent patients has been proposed as a contributing factor to increased unprotected sex and subsequent STI’s: this link is as yet unproven [10–15]. The CDC reports the highest absolute rates of syphilis in those aged 30–39, our data however, identifies that the highest rate of syphilis in our patients occurs between 40 and 49 years of age [4]. This may be attributable to successful ART allowing HIV-infected patients to live longer, remain in good health and be involved in ongoing sexual relationships [12]. Less attention towards safer sex practices and lower uptake of STI testing among the older cohort may also be contributing factors [21].

Coinfection with STIs including *N. gonorrhoeae*, *C. trachomatis*, and HCV has shown to have implications for HIV treatment and transmission [22–25]. With the knowledge that over 40% of these patients had prior or current STIs including *N. gonorrhoeae* and *C. trachomatis*, regular screening is recommended as it has been shown to increase early detection and prevent subsequent transmission [22, 17, 24, 25].

Over one quarter of the syphilis infections were repeat infections. The frequency of repeat infections is increasing over time, while in 2009, only 14% of the incident infections were repeat episodes by 2016, 44% were repeat infections. This is mirrored by a study published by Phipps et al. that evaluated repeat syphilis infections between 2001 and 2002 among MSM in San Francisco and found that of 642 patients with diagnosis of syphilis, 63% were co-infected with HIV and 6.7% were repeat infections within one year of prior syphilis diagnosis [6].

Of Public Health concern those with repeat infections were more likely to have detectable HIV viremia when compared to those with initial syphilis episodes. This subpopulation requires intensive investigation as repeat syphilis infection indicates continued high risk behaviors and in the setting of also having HIV viral load > 1000 copies/mL increases the risk of HIV transmission [4, 11–15]. As such, they may benefit most from comprehensive preventative programs, including counselling and education, increased frequency of screening, outreach interventions and possibly STI pre-exposure prophylaxis [4, 25–28].

We found that patients at the time of their syphilis episode were nearly twice as likely to not be on ART as those with HIV but negative for syphilis. Furthermore, in 19% of cases, the patients diagnosed with syphilis had HIV viremia > 1000 copies/mL potentially increasing their risk of HIV transmission to uninfected partners [11, 15]. HIV PrEP has had success in the prevention of HIV transmission, and its use in the context of the current syphilis epidemic is being evaluated. Bolan et al. undertook a pilot study in 2015, providing doxycycline prophylaxis to reduce incident syphilis among HIV-infected MSM. Their results showed that STI PrEP with doxycycline was associated with a decreased incidence of *N. gonorrhoeae*, *C. trachomatis* and syphilis in this population [27].

Post exposure prophylaxis with doxycycline has also been shown to reduce the overall incidence in bacterial STI’s in HIV-infected MSM, however the long-term benefit is still unknown and therefore antibiotic prophylaxis for STI’s is currently not recommended in guidelines [28]. This may be an avenue for primary prevention of syphilis in HIV-positive individuals in regular care and characterized to be high risk for syphilis infection.

Our study has limitations. We only address patients who are accessing care. The incident rates reported may be an under estimate as they do not include individuals infected but lost to follow up and do not include those who were diagnosed with syphilis in Alberta, however subsequently moved out of province, resulting in inadequate follow up data. Although the study population, while comprehensive and representing a regional Canadian perspective, is from a single regional area and may not be representative populations elsewhere that have different rates of unprotected sexual activity and both prevalent HIV and syphilis infections. In addition, access to care varies between centers and populations and our rates and identification methods may not precisely match others. In our population, however care for both HIV and STI care is centralized and well-coordinated, allowing for detailed analyses.

## Conclusions

Increasing incident syphilis infections among the HIV-infected population in care is an emerging public health concern. Highlighted is the importance of a regular syphilis screening program as half of the syphilis infections identified were asymptomatic, diagnosis prompted not only earlier treatment but also identified patients with high risk sexual behaviors. Regular safer sex counselling in our program has not been effective in limiting syphilis acquisition in those successfully engaged in HIV care. A number of risk factors among HIV-infected populations were identified, including; MSM, prior alcohol abuse, prior recreational drug use and prior syphilis episode. Intensified and novel interventions will be required for those at high risk if the negative consequences of syphilis are to be avoided and its spread curtailed.

## Additional file

Additional file 1: **Table S1.** Characteristics of HIV+ patients regularly followed at the Southern Alberta Clinic between 1/1/2006 and 12/31/2016 comparing patients in four groups: syphilis positive (reference group), repeat syphilis positive, syphilis positive not on ART, syphilis positive not HIV Virologically suppressed (VL > 40 copies/mL). ^1^Indigenous includes Aboriginal, Metis and Inuit; ACB includes African, Caribbean, Black; Other includes IndoAsian, Hispanic, East Asian, and other. ^2^MSM = self-reported men who have sex with men identification; HET = self-reported heterosexual identification; PWID = self-reported intravenous drug use identification; Other HIV Risk factor behavior includes: blood transfusions, hemophiliac, neonatal, postnatal infection, unknown or not reported. ^3^History of Cigarette Smoking-Current or Past; History of Alcohol Abuse- > 14 drinks/week or binge drinking; History of Recreational Drug Use –Current or Past; History of Intimate Partner Violence-Current or Past. (DOCX 24 kb)

## References

[1] World Health Organization. (2012) Global incidence and prevalence of selected curable sexually transmitted infections – 2008. http://www.who.int/reproductivehealth/publications/rtis/stisestimates/en/. Accessed 2 Nov 2016.

[2] Trotten S, Maclean R, Payne E. Infectious syphilis in Canada: 2003–2012, CCDR. Center for Communicable Diseases and Infection Control, Public Health Agency of Canada. CCDR. 2015;41(2). 10.14745/ccdr.v41i02a03 PMC5864306 29769929

[3] Alberta Government. Sexually transmitted infections have reached outbreak levels in Alberta. 2016 Apr 26. https://www.alberta.ca/release.cfm?xID=4163947EECDEA-024A-0016-6E2A2E61E3978E3E. Accessed 2 Nov 2016.

[4] Cope AB, Crooks AM, Chin T, Kuruc JD, McGee KS, Eron JJ (2014). Incident sexually transmitted infection as a biomarker for high risk sexual behaviour following diagnosis with acute HIV. Sex Transm Dis.

[5] Palacios R, Jimenez-Onate F, Aguilar M, Galindo MJ, Ocampo A (2007). Berenguer et al. impact of syphilis infection on HIV viral load and CD4 cell counts in HIV-infected patients. J Acquir Immune Defic Syndr.

[6] Phipps W, Kent CK, Kohn R, Klausner JD (2009). Risk factors for repeat syphilis in men who have sex with men, San Francisco. Sex Trans Dis.

[7] Kofoed K, Gerstoft J, Mathiesen LR, Benfield T (2006). Syphilis and human immunodeficiency virus (HIV)-1 coinfection: influence on CD4 T-cell count, HIV-1 viral load, and treatment response. Sex Transm Dis.

[8] Sadiq ST, McSorley J, Copas AJ, Bennett J, Edwards SJ, Kaye S (2005). The effects of early syphilis on CD4 counts and HIV-1 RNA viral loads in blood and semen. Sex Transm Infect.

[9] Hall HI, Holtgrove DR, Maulsby C (2012). HIV transmission rates from persons living with HIV who are aware and unaware of their infection. AIDS.

[10] Braun DL, Marzel A, Steffens D, Schreiber PW, Grube C, Scherrer AU, et al. High rates of subsequent asymptomatic STIs and risky sexual behavior in patients initially presenting with primary HIV-1 infection. Clin Infect Dis Published Online First: 5 October 2017. doi:10.1093/cid/cix873. 10.1093/cid/cix873 29028966

[11] R. v Mabior, 2012 SCC 47, [2012] 2 S.C.R. 584. https://scc-csc.lexum.com/scc-csc/scc-csc/en/item/10008/index.do. Accessed: 1 Dec 2017.

[12] Ciesielski CA (2003). Sexually transmitted diseases in men who have sex with men: an epidemiologic review. Curr Infect Dis Rep.

[13] Kalichman SC, Pellowski J, Turner C (2011). Prevalence of sexually transmitted co-infections in people living with HIV/AIDS: systematic review with implications for using HIV treatments for prevention. Sex Transm Infect.

[14] Shilaih M, Marzel A, Braun DL, Scherrer AU, Kovari H, Young J (2017). Factors associated with syphilis incidence in the HIV-infected in the era of highly active antiretrovirals. Medicine.

[15] Quinn TC, Wawer MJ, Sewankambo et al. Viral load and heterosexual transmission of human immunodeficiency virus type 1. NEJM, 2000;342: 921–929. 10.1056/NEJM200003303421303 10738050

[16] Newman L, Rowley J, Vander Hoorn S, Wijesooriya NS, Unemo M, Low N, et al. Global estimates of the prevalence and incidence of four curable sexually transmitted infections in 2012 based on systematic review and global reporting. PLoS One. 2015;10(12):12–20. 10.1371/journal.pone.0143304 PMC4672879 26646541

[17] Centers for Disease Control and Prevention. (2015) Sexually Transmitted Diseases: Syphilis. Available at: http://www.cdc.gov/std/. Accessed 2 Nov 2016.

[18] Bolu OO, Lindsey C, Kamb ML, Kent C, Zenilman J, Douglas JM (2004). Is HIV/sexually transmitted disease prevention counseling effective among vulnerable populations: a subset analysis of data collected for a randomized, controlled trial evaluating counseling efficacy (project RESPECT). Sex Transm Dis.

[19] Richardson JL, Milam J, McCutchan A, Stoyanoff S, Bolan R, Weis J (2004). Effect of brief safer-sex counseling by medical providers to HIV-1 seropositive patients: a multi-clinic assessment. AIDS.

[20] Rietmeijer CA (2007). Risk reduction counselling for prevention of sexually transmitted infections: how it works and how to make it work. Sex Transm Infect.

[21] Fleming DT, Wasserheit JN (1999). From epidemiological synergy to public health policy and practice: the contribution of other sexually transmitted diseases to sexual transmission of HIV infection. Sex Transm Infect.

[22] Baffi CW, Aban I, Willig JH, Agrawal M, Nugavero MJ, Bachman LH (2010). New syphilis cases and concurrent STI screening in a southeastern U.S. HIV clinic: a call to action. AIDS Patient Care STDs.

[23] Hu Q, Xu J, Zou HC, Liu J, Ding H, Qian H (2014). Risk factors associated with prevalent and incident syphilis among an HIV-infected cohort in Northeast China. BMC Infect Dis.

[24] Cantor AG, Pappas M, Daeges M, Nelson HD (2016). Screening for syphilis: updated evidence report and systematic review for the US preventive service task force. JAMA.

[25] Winston A, Hawkins D, Mandalia S, Boag F, Azadian B, Asboe D (2003). Is increased surveillance for asymptomatic syphilis in an HIV outpatient department worthwhile?. Sex Transm Infect.

[26] Chaulk P, Ravikiran M, Morgan J, Pearl ML, Matus S, Blythe D, et al. Notes from the Field: Repeat Syphilis Infection and HIV Coinfection Among Men Who Have Sex with Men- Baltimore, Maryland, 2010–2011. Centers for Disease Control and Prevention (CDC). http://www.cdc.gov/mmwr/preview/mmwrhtml/mm6232a4.htm. Accessed 2 Nov 2016.

[27] Bolan RK, Beymer MR, Weiss RE, Flynn RP, Leibowitz AA, Klausner JD (2015). Doxycycline prophylaxis to reduce incident syphilis among HIV-infected men who have sex with men who continue to engage in high risk sex: a randomized, controlled pilot study. Sex Transm Dis.

[28] Molina JM, Charreau I, Chidiac C, Pialoux G, Cua E, Delaugerre C, et al. On demand post exposure prophylaxis with doxycycline for MSM enrolled in a PrEP Trial [abstract]. In: CROI; 2017 Feb 13–16; Seattle, WA. IAS-USA. Abstract 91LB.

