# Supplementary material for: Increasing incidence of syphilis among patients engaged in HIV care in Alberta, Canada: a retrospective clinic-based cohort study
Source: BMC Infect Dis. 2018 Mar 13;18:125. doi: 10.1186/s12879-018-3038-4 (PMC5851255; doi:10.1186/s12879-018-3038-4)
Supplement: Supplementary file 1 — Table S1. Characteristics of HIV+ patients regularly followed at the Southern Alberta Clinic between 1/1/2006 and 12/31/2016 comparing patients in four groups: syphilis positive (reference group), repeat syphilis positive, syphilis positive not on ART, syphilis positive not HIV Virologically suppressed (VL > 40 copies/mL). 1Indigenous includes Aboriginal, Metis and Inuit; ACB includes African, Caribbean, Black; Other includes IndoAsian, Hispanic, East Asian, and other. 2MSM = self-reported men who have sex with men identification; HET = self-reported heterosexual identification; PWID = self-reported intravenous drug use identification; Other HIV Risk factor behavior includes: blood transfusions, hemophiliac, neonatal, postnatal infection, unknown or not reported. 3History of Cigarette Smoking-Current or Past; History of Alcohol Abuse- > 14 drinks/week or binge drinking; History of Recreational Drug Use –Current or Past; History of Intimate Partner Violence-Current or Past. (DOCX 24 kb) [file 12879_2018_3038_MOESM1_ESM.docx]

**Additional file 1: Table S1: Characteristics of HIV+ patients regularly followed at the Southern Alberta Clinic between 1/1/2006 and 12/31/2016 comparing patients in four groups: syphilis positive (reference group), repeat syphilis positive, syphilis positive not on ART, syphilis positive not HIV Virologically suppressed (VL>40 copies/mL).**

| **Characteristic** | **All Episodes of Syphilis Positive** | **Repeat Episodes of Syphilis Positive** | **P -value** | **Not on ART** | **P- value** | **Not Virologically suppressed** | **P -value** |
| --- | --- | --- | --- | --- | --- | --- | --- |
| **N** (%) | 194 | 36 |  | 43 |  | 64 |  |
| **Age at HIV diagnosis** (years)  **Mean** (Range) | 35 (16-69) | 34 (18-58) | 0.552 | 38 (21-59) | 0.068 | 32 (16-58) | 0.027 |
| **Age group:** |  |  | 0.857 |  | 0.216 |  | 0.202 |
| <30 | 75 (38.7) | 16 (44.4) |  | 21 (48.9) |  | 32 (50.0) |  |
| 30-39 | 66 (34.0) | 11 (30.6) |  | 17 (39.5) |  | 21 (32.8) |  |
| 40-49 | 37 (19.1) | 6 (13.6) |  | 3 (7.0) |  | 7 (10.9) |  |
| >50 | 16 (8.2) | 3 (8.3) |  | 2 (4.7) |  | 4 (6.2) |  |
| **Gender** |  |  | **0.007** |  | **0.020** |  | **0.002** |
| Male | 183 (94.3) | 35 (97.2) |  | 40 (93.0) |  | 60 (93.8) |  |
| Female | 11 (5.6) | 1 (2.8) |  | 1 (7.0) |  | 4 (6.2) |  |
| Transgender | 0 (0) | 0 (0) |  | 0 (0) |  | 0 (0) |  |
| **Self-reported Ethnicity^1^** |  |  | **0.001** |  | 0.135 |  | **0.04** |
| Caucasian | 140 (72.2) | 27 (75.0) |  | 33 (76.7) |  | 49 (76.6) |  |
| Indigenous | 6 (3.1) | 1 (2.8) |  | 1 (2.3) |  | 2 (3.1) |  |
| ACB | 24 (12.4) | 1 (2.8) |  | 5 (11.6) |  | 7 (10.9) |  |
| Other | 24 (12.4) | 7 (19.4) |  | 4 (9.3) |  | 6 (9.4) |  |
| **Most Likely HIV Exposure Categor**y^2^ |  |  | **<0.001** |  | **0.004** |  | **<0.001** |
| MSM | 145 (74.7) | 33 (91.7) |  | 29 (67.4) |  | 47 (73.4) |  |
| HET | 14 (7.2) | 1 (2.8) |  | 3 (7.0) |  | 3 (4.7) |  |
| PWID | 30 (15.5) | 2 (5.6) |  | 10 (23.3) |  | 13 (20.3) |  |
| Other | 5 (2.6) | 0 (0) |  | 1 (2.3) |  | 1 (1.6) |  |
| **Cofactors**^3^ |  |  |  |  |  |  |  |
| History of Smoking | 110 (56.7) | 18 (50.0) | 1.000 | 25 (58.1) | 0.414 | 38 (59.4) | 0.212 |
| History of Alcohol Abuse | 56 (28.9) | 9 (25.0) | 0.380 | 14 (32.6) | **0.024** | 21 (32.8) | **0.004** |
| History of Recreational Drug Use | 87 (44.8) | 17 (47.2) | 0.064 | 19 (44.2) | 0.104 | 27 (42.2) | 0.089 |
| History of Intimate Partner Violence | 41 (21.1) | 6 (16.7) | 0.220 | 8 (18.6) | 0.276 | 14 (21.9) | 0.418 |

^1^Native Canadian includes Aboriginal, Metis and Inuit; ACB includes African, Caribbean, Black; Other includes IndoAsian, Hispanic, East Asian, and other

^2^MSM=self-reported men who have sex with men identification; HET=self-reported heterosexual identification; PWID=self-reported intravenous drug use identification; Other HIV Risk factor behavior includes: blood transfusions, hemophiliac, neonatal, postnatal infection, unknown or not reported.

^3^History of Cigarette Smoking-Current or Past; History of Alcohol Abuse->14 drinks/week or binge drinking; History of Recreational Drug Use –Current or Past; History of Intimate Partner Violence-Current or Past.
